# Defective insulin receptor signaling in hPSCs skews pluripotency and negatively perturbs neural differentiation

**DOI:** 10.1016/j.jbc.2021.100495

**Published:** 2021-03-03

**Authors:** Adrian Kee Keong Teo, Linh Nguyen, Manoj K. Gupta, Hwee Hui Lau, Larry Sai Weng Loo, Nicholas Jackson, Chang Siang Lim, William Mallard, Marina A. Gritsenko, John L. Rinn, Richard D. Smith, Wei-Jun Qian, Rohit N. Kulkarni

**Affiliations:** 1 Section of Islet Cell and Regenerative Biology, Department of Medicine, Joslin Diabetes Center, Brigham and Women’s Hospital, Harvard Medical School, and Harvard Stem Cell Institute, Boston, Massachusetts, USA; 2 Stem Cells and Diabetes Laboratory, Institute of Molecular and Cell Biology, Proteos, Singapore, Singapore; 3 Department of Biochemistry and Department of Medicine, Yong Loo Lin School of Medicine, National University of Singapore, Singapore, Singapore; 4 School of Biological Sciences, Nanyang Technological University, Singapore, Singapore; 5 Department of Stem Cell and Regenerative Biology, Harvard University, and Broad Institute of MIT, Cambridge, Massachusetts, USA; 6 Biological Sciences Division, Pacific Northwest National Laboratory, Richland, Washington, USA

**Keywords:** human, insulin receptors, signaling, stem cells, differentiation, pluripotency, cell fate specification, neural lineage, AKT, ERK1/2, ECM, extracellular matrix, hESC, human embryonic stem cell, hPSC, human pluripotent stem cell, IGF, insulin-like growth factor, IR, insulin receptor, KOSR, KnockOut Serum Replacement

## Abstract

Human embryonic stem cells are a type of pluripotent stem cells (hPSCs) that are used to investigate their differentiation into diverse mature cell types for molecular studies. The mechanisms underlying insulin receptor (IR)-mediated signaling in the maintenance of human pluripotent stem cell (hPSC) identity and cell fate specification are not fully understood. Here, we used two independent shRNAs to stably knock down IRs in two hPSC lines that represent pluripotent stem cells and explored the consequences on expression of key proteins in pathways linked to proliferation and differentiation. We consistently observed lowered pAKT in contrast to increased pERK1/2 and a concordant elevation in pluripotency gene expression. ERK2 chromatin immunoprecipitation, luciferase assays, and ERK1/2 inhibitors established direct causality between ERK1/2 and OCT4 expression. Of importance, RNA sequencing analyses indicated a dysregulation of genes involved in cell differentiation and organismal development. Mass spectrometry–based proteomic analyses further confirmed a global downregulation of extracellular matrix proteins. Subsequent differentiation toward the neural lineage reflected alterations in SOX1^+^PAX6^+^ neuroectoderm and FOXG1^+^ cortical neuron marker expression and protein localization. Collectively, our data underscore the role of IR-mediated signaling in maintaining pluripotency, the extracellular matrix necessary for the stem cell niche, and regulating cell fate specification including the neural lineage.

Human embryonic stem cells (hESCs) are prototypical human pluripotent stem cells (hPSCs) widely used for studying pluripotency and differentiation (1). Pluripotency factors (2) as well as Activin/Nodal and fibroblast growth factor (FGF) signaling (3) define the characteristics of hPSCs. In recent years, the use of KnockOut Serum Replacement (KOSR) and defined media for culturing hPSCs such as StemPro, mTeSR1, and Essential 8 (E8) has shed light on the extracellular components necessary for maintaining human pluripotency and metabolic homeostasis.

These hPSC media that contain supraphysiological levels of insulin and insulin-like growth factors (IGFs) suggest a requirement for insulin and/or IGF-I receptors in regulating hPSCs (4). In general, insulin binds insulin receptor (IR) isoforms IR-A (-exon 11) or IR-B (+exon 11), IGF-II binds IR-A or IGF1R, and IGF-I binds IGF1R (5). Indeed, the IGF-I analog heregulin-1β supports hESC proliferation and self-renewal (6), whereas IGF-II alone is sufficient to maintain hESC cultures (7). Conversely, blockade of IGF1R decreases survival of hESCs (7) and promotes differentiation (6). Beyond pluripotency, the knockout of IR in the mouse brain has also been reported to lead to neuronal defects that contribute to neurological disorders (8). Although most studies to date have focused on IGF1R signaling in regulating human pluripotency, the precise role of IR in hPSCs and their differentiated progeny has received poor attention.

To directly address the significance of IR in pluripotency and hPSC identity, we used two independent shRNAs to stably knock down IR in two different hPSC lines and interrogated the consequences on insulin/IGF-I signaling pathways (9). Knock down of IR decreased basal pAKT and reciprocally increased pERK1/2 levels. Next, genome-wide RNA sequencing (RNA-Seq) and mass spectrometry (MS)-based proteomics analyses in shIR-hPSCs revealed an increased expression of numerous pluripotency genes (OCT4, SOX2, DPPA4, and LIN28). The compensatory increase in pERK1/2 could possibly account for the elevated pluripotency gene expression since ERK2 has been reported to bind proximal to the promoter of pluripotency genes (10). ERK2 chromatin immunoprecipitation, luciferase assays, and ERK1/2 inhibitors together establish direct causality between ERK1/2 and OCT4 expression. It is striking that RNA-Seq and proteomics analyses revealed a dysregulation of germ layer cell fate specification genes and an unexpected global downregulation of extracellular matrix (ECM) genes. When these shIR-hPSCs were differentiated toward the neural lineage, we observed aberrant neuroectoderm and cerebral organoid development. Together, these results demonstrate the importance of IR-mediated signaling in the maintenance of pluripotency, the ECM which contributes to the hPSC niche (11), and long-lasting effects on tissue and neural differentiation.

## Results

### Knock down of IR in hPSCs perturbs insulin/AKT/ERK1/2 signaling pathways

Consistent with previous reports (12, 13), we detected high levels of human insulin in Dulbecco's modified Eagle's medium (DMEM)/F-12/20% KOSR (∼1200 ± 175 nM) and mTeSR1 media (∼1407 ± 128 nM). Although the rationale for adding >100× physiological concentration of recombinant insulin/IGFs to hPSC media is unclear, one implication is that insulin/IGF signaling is important for the maintenance of hPSCs.

**Figure 1:**
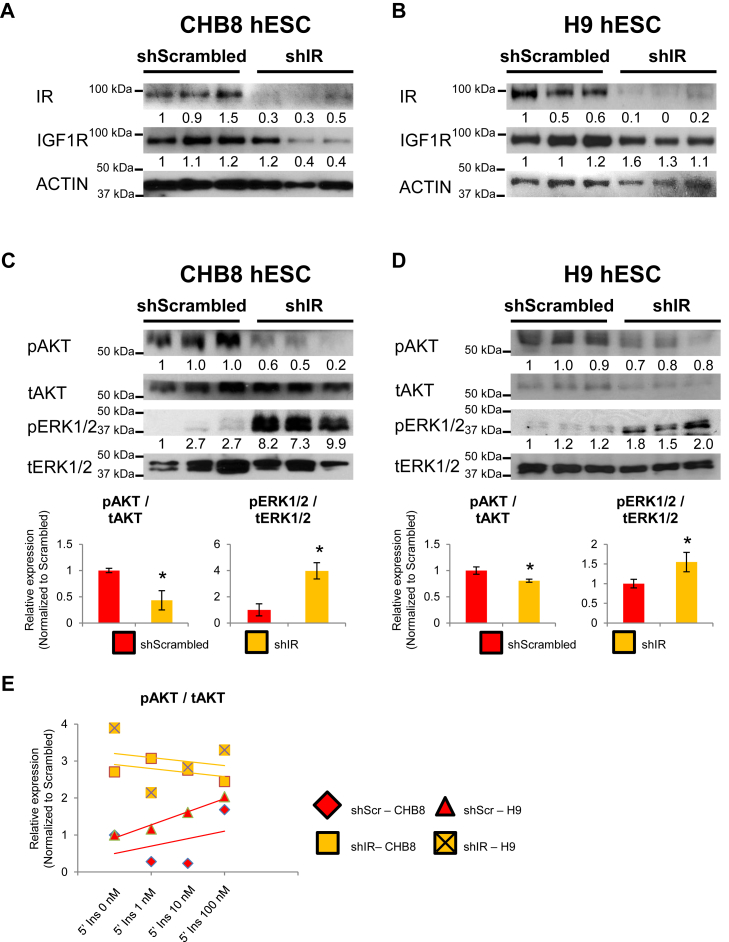
**Knockdown of IR in CHB8 and H9 hESCs perturbs the insulin signaling pathway.** Western blot analyses for IR, IGF1R, and ACTIN protein levels in shScr and shIR in two different hESCs: (*A*) CHB8 and (*B*) H9 hESCs. Western blot analyses for pAKT, tAKT, pERK1/2, and tERK1/2 protein levels in shScr- and shIR- (*C*) CHB8 and (*D*) H9 hESCs. pAKT, tAKT, pERK1/2, and tERK1/2 Western blot bands were quantitated and represented as a ratio to depict pAKT and pERK1/2 signaling levels, respectively (*lower panels*). At least two independent experiments have been performed. All *error bars* indicate standard deviation of three biological replicates. *Asterisk* (∗) indicates *p* < 0.05 compared with shScr-hPSCs (Student’s *t* test). *E*, pAKT and tAKT Western blot bands were quantitated and represented as a ratio to depict pAKT signaling levels upon stimulation of shScr- and shIR-hPSCs with exogenous insulin for 5 min. Data from additional independent IR knocked down clones is presented in Fig S1. hESC, human embryonic stem cell; hPSC, human pluripotent stem cell; IR, insulin receptor.

To directly investigate the role of IR signaling in mediating human pluripotency, we stably knocked down IR in two different hESC lines (CHB8 and H9). Among the four shRNA constructs (Sigma) we tested, the short hairpins that target the CACTGATTACTTGCTGCTCT sequence in exon 2 and GTGCTGTATGAAGTGAGTTA sequence in exon 13 of IR gene (*INSR*) resulted in a reproducible knockdown of IR transcript and protein expression. Of a total of 52 clones (35 for CHB8 and 17 for H9) that were screened both at the transcript and protein levels, we obtained two independent IR knocked down clones in CHB8 (shIR-CHB8) and three in H9 hESCs (shIR-H9). QPCR and Western blot analyses confirmed the knock down of IR in CHB8 (Fig. 1*A* and Fig. S1*A*) and H9 hESCs (Fig. 1*B* and Fig. S1*B*). Of interest, knock down of IR resulted in a concomitant decrease in IGF1R protein expression (Fig. 1, *A* and *B*).

Next, upon IR knockdown, we observed that IRS-1 and p85α protein expression was marginally decreased, whereas IRS-2 protein expression either increased or did not change (Fig. S1, *C*–*E*). Notwithstanding the mild changes in expression of the proximal adaptor proteins, we observed a drastic decrease in pAKT^S473^ and a surprising reciprocal increase in pERK1/2^T202/Y204^ signaling (Fig. 1, *C* and *D*). Western blot analyses on additional independent IR knocked down clones in H9 hESCs, generated using different shRNA constructs, reflected similar changes in protein expression (Fig. S1*E*). Together, these data indicate that IR/AKT/ERK1/2 signaling is active in hPSCs and the loss of IR modulates PI3K/AKT and MEK/ERK signaling pathways in a reciprocal manner, which may, in turn, impact the hPSC state.

### shIR-hPSCs are insensitive to insulin stimulation at the pAKT level

To determine if the shIR-hPSCs continued to respond normally to ligand, we starved the hPSCs for 24 h in DMEM/F-12 + 0.5% bovine serum albumin (BSA) + 10 ng/ml Activin + 12 ng/ml FGF2 (3) (minimal pluripotency-sustaining conditions devoid of undefined factors that would activate insulin signaling) before stimulating them with increasing doses of human insulin. The scrambled controls (shScr-hPSCs) exhibited a dose-dependent increase in pAKT^S473^ upon insulin stimulation (Fig. 1*E*), indicating primary activation of the PI3K/AKT signaling pathway in the maintenance of self-renewal and pluripotency. In contrast, shIR-hPSCs lost their responsiveness to exogenous insulin stimulation (Fig. 1*E*).

### shIR-hPSCs exhibit compensatory increase in pluripotency gene expression

**Figure 2:**
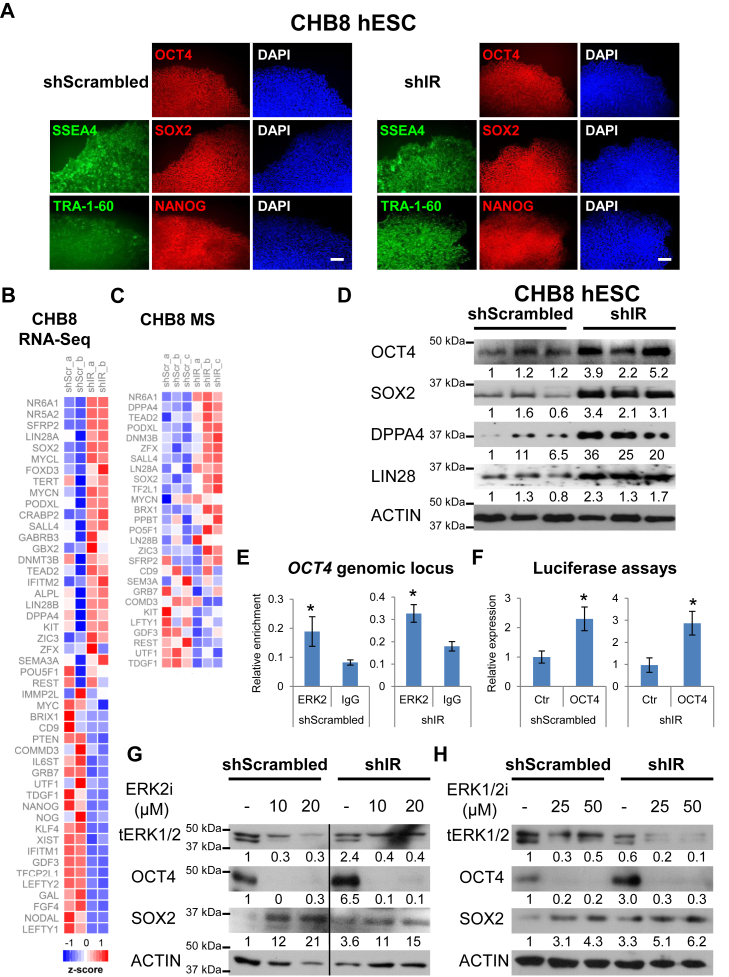
**shIR-hPSCs exhibit increased pluripotency gene expression.***A*, immunostaining for OCT4, SOX2, NANOG, SSEA-4, and TRA-1-60 pluripotency markers in shScr- and shIR-CHB8 hESCs. Scale bar: 200 μm. *B*, heat map of RNA-Seq analyses of genes involved in pluripotency from shScr- and shIR-CHB8 hESCs (upregulation in *red*, downregulation in *blue*). *C*, heat map showing mass spectrometry analyses of proteins involved in pluripotency from shScr- and shIR-CHB8 hESCs (upregulation in *red*, downregulation in *blue*). *D*, Western blot analyses for OCT4, SOX2, DPPA4, LIN28, and ACTIN protein levels in shScr- and shIR-CHB8 hESCs. *E*, ERK2 binds onto *OCT4* genomic locus in a ChIP assay in shScr- and shIR-H9 hESCs. *F*, ERK2-bound *OCT4* genomic locus is transcriptionally active in a luciferase assay in shScr- and shIR-CHB8 hESCs. The inhibition of (*G*) ERK2 or (*H*) ERK1/2 with two different inhibitors results in the abolishment of OCT4 expression but not SOX2 in shScr- and shIR-H9 hESCs. All error bars indicate standard deviation of three replicates. *Asterisk* (∗) indicates *p* < 0.05 (Student’s *t* test). Data from additional independent IR knocked down clones is presented in Fig S2. hESC, human embryonic stem cell; hPSC, human pluripotent stem cell; IR, insulin receptor.

Next, to determine if the knock down of IR in hPSCs affected the hPSC state, we immunostained both shScr- and shIR-hPSCs for OCT4, SOX2, NANOG, SSEA4, and TRA-1-60 and confirmed that they remain pluripotent (Fig. 2*A*). We also differentiated them into the three germ layers *in vivo via* teratoma assays (Fig. S2*A*) and performed *in vitro* directed differentiation (Fig. S2*B*) to definitively demonstrate their pluripotency. We next undertook a systems biology approach to gain further insights into the impact of IR knock down on hPSCs. RNA-Seq performed on shIR-CHB8 hESCs indicated that *INSR* transcript was knocked down without affecting *IGF1R* transcripts (Table S1), suggesting that the short hairpins targeting *INSR* transcript are specific and that the decrease in IGF1R protein expression (Fig. 1, *A* and *B* and Fig. S1*E*) is secondary to the loss of IR protein expression. Proteomics analyses further confirmed that IR protein was knocked down in shIR-CHB8 hESCs (Table S2).

RNA-Seq analyses performed on shIR-CHB8 hESCs revealed an interesting trend of upregulation of numerous pluripotency genes (Fig. 2*B*, Fig. S2*C* and Table S1). Proteomics analyses revealed similar changes in both CHB8 (Fig. 2*C* and Fig. S2*D*) and H9 (Fig. S2, *E* and *F*) hESCs (Tables S2 and S3). Western blot analyses confirmed that the knock down of IR resulted in an upregulation of OCT4, SOX2, DPPA4, and LIN28 protein expression both in CHB8 (Fig. 2*D*) and H9 (Fig. S2, *G* and *H*) hESCs.

Phosphoproteomics analyses performed on shIR-H9 hESCs confirmed an increased phosphorylation of ERK1^Y204^ and ERK2^Y187^ (Table S4), corroborating our observations on increased pERK1/2^T202/Y204^ in shIR-hPSCs (Fig. 1, *C* and *D* and Fig. S1*E*). These data gain significance since ERK2 has been reported to bind to promoter/enhancer regions of pluripotency genes *OCT4*, *SOX2*, *DPPA4*, *LIN28A*, *SALL4*, and *DNMT3B* (10). Furthermore, OCT4, SOX2, and SALL4 all contain putative ERK phosphorylation sites among which ERK2 was confirmed to phosphorylate OCT4, thereby directly linking ERK signaling with pluripotency (14). Therefore, we hypothesized that the loss of IR in hPSCs led to a compensatory increase in pERK1/2, which in turn upregulated the expression of pluripotency genes to maintain the hPSC state.

To establish direct causality between ERK1/2 and pluripotency, we first performed ERK2 chromatin immunoprecipitation (ChIP) qPCR analyses on promoter/enhancer regions of pluripotency genes *OCT4*, *SOX2*, *DPPA4*, *LIN28A*, *SALL4*, and *ZFX* as indicated by Goke *et al.* (2013) (10). Among these genomic loci, we only found ERK2 to consistently bind onto the *OCT4* enhancer region (Fig. 2*E*). To confirm that the *OCT4* enhancer region bound by ERK2 is transcriptionally active, we cloned the promoter/enhancer regions of *OCT4*, *SOX2*, and *DPPA4* into luciferase assay vectors. Among these three pluripotency genes, indeed we found the *OCT4* enhancer region to be transcriptionally active (Fig. 2*F*). To further demonstrate that the direct interaction between ERK1/2 and *OCT4* genomic loci is functionally active, we used two different ERK1/2 inhibitors (instead of MEK inhibitors) on shScr- and shIR-hPSCs and observed that the inhibition of ERK1/2 reproducibly abolished the protein expression of OCT4 but not that of SOX2 (Fig. 2, *G* and *H*). The knockdown of ERK1/2 *via* shRNAs was unfortunately unsuccessful across multiple attempts.

To identify other pluripotency genes that could be directly phosphorylated and regulated by pERK1/2, we performed phosphoproteomics analyses on shIR-CHB8 and shIR-H9 hESCs. NR6A1, SALL4, and DPPA4 exhibited increased phosphorylation (*p* < 0.05) in shIR-CHB8 hESCs (Table S5), whereas DPPA4 and UTF1 exhibited increased phosphorylation (*p* < 0.05) in shIR-H9 hESCs (Table S4). DPPA4 distinctly exhibited increased phosphorylation (*p* < 0.05) in both hESCs (Tables S4 and S5). Taken together, our data suggest that the increased pERK1/2 due to loss of IR in hPSCs phosphorylates and increases the expression of pluripotency genes to maintain the hPSC state.

### shIR-hPSCs exhibit perturbations in cell fate markers and decreased ECM gene expression

**Figure 3:**
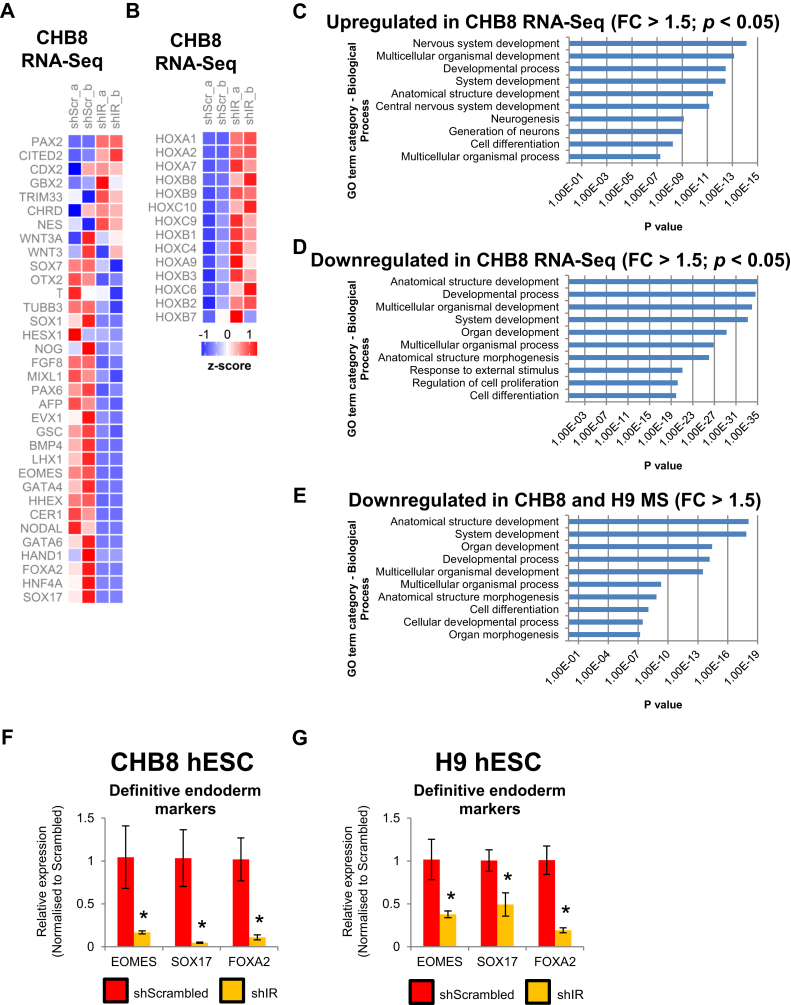
**Knockdown of IR in hPSCs perturbs cell fate commitment balance.** Heat map of RNA-Seq analyses showing (*A*) germ layer and (*B*) *HOX* gene expression from shScr- and shIR-CHB8 hESCs (upregulation in *red*, downregulation in *blue*). GO analysis of differentially (*C*) upregulated and (*D*) downregulated genes (RNA-Seq data) in shIR-CHB8 hESCs. *E*, GO analysis of differentially downregulated genes (MS data) in both shIR-CHB8 and shIR-H9 hESCs. GO terms are indicated along the *y*-axis and the *p* values for significance of enrichment for the top 10 Gene Ontology terms along the *x*-axis. Expression of *EOMES*, *SOX17*, and *FOXA2* transcripts in shScr- and shIR- (*F*) CHB8 and (*G*) H9 hESCs. All *error bars* indicate standard deviation of three biological replicates. *Asterisk* (∗) indicates *p* < 0.05 compared with shScr-hPSCs (Student’s *t* test). hESC, human embryonic stem cell; hPSC, human pluripotent stem cell; IR, insulin receptor.

Since shIR-hPSCs exhibited increased pERK1/2 and gene expression of pluripotency factors, we next sought to determine if the increased expression of pluripotency genes modulated cell fate specification markers, in line with reports that increased pERK1/2 can lead to increased differentiation (15). Initial triage of RNA-Seq data from shIR-CHB8 hESCs (fold change > 1.5; *p* < 0.05) *via* Gene Ontology (GO) analyses indicated an upregulation of genes linked to nervous system development and neurogenesis (Fig. 3, *A*–*C*). This correlated with the increased expression of all Hox genes (majorly expressed in the vertebrate nervous system) detected in the RNA-Seq data (among 20,469 genes) (Fig. 3*B*). In addition, a majority of the most differentially upregulated genes in shIR-hPSCs were related to the neural lineage (Table S1).

GO analyses indicated that multiple biological processes were both up- and downregulated in the RNA-Seq data (Fig. 3, *C* and *D*). Among the early germ layer specification genes, we observed that indeed numerous neuroectodermal genes such as *PAX2*, *GBX2*, *CHRD*, and *NES* and numerous *HOX* genes were upregulated, whereas most of the mesodermal and endodermal genes were strikingly downregulated in shIR-hPSCs (Fig. 3, *A* and *B*). GO analyses performed on downregulated proteins in the proteomics data from both shIR-CHB8 and shIR-H9 hESC lines (fold change > 1.5) (Fig. 3*E*) corroborated the RNA-Seq data (Fig. 3*D*). QPCR analyses then confirmed that some of the most downregulated genes (mostly endodermal) such as *EOMES*, *SOX17*, and *FOXA2* were severely suppressed in shIR-hPSCs (Fig. 3, *F* and *G*). Thus, knockdown of IR in hPSCs causes an imbalance in germ layer specification genes, likely owing to an increase in phosphorylation coupled with total protein expression of numerous pluripotency genes.

The knock down of IR in hPSCs also distinctly resulted in a global downregulation of ECM gene expression. RNA-Seq data indicated that a majority of the collagen (*COL*), keratins (*KRT*), vitronectin (*VTN*), fibronectin (*FN1*), laminins (*LAM*), and prolyl 4-hydroxylase (*P4H*) genes were dramatically downregulated in shIR-hPSCs (Fig. S3*A*). Proteomics data in shIR-CHB8 and shIR-H9 hESCs further reflected this global downregulation of ECM proteins (Fig. S3, *B* and *C*). QPCR analyses on *FN1*, *COL1A1*, and *KRT19* genes in both shIR-CHB8 and shIR-H9 hESCs (Fig. S3*D*) and Western blot analyses on FN1, COL1, and P4HB (Fig. S3*E*) provided corroborative independent validations.

### shIR-hPSCs exhibit aberrant neural lineage differentiation

**Figure 4:**
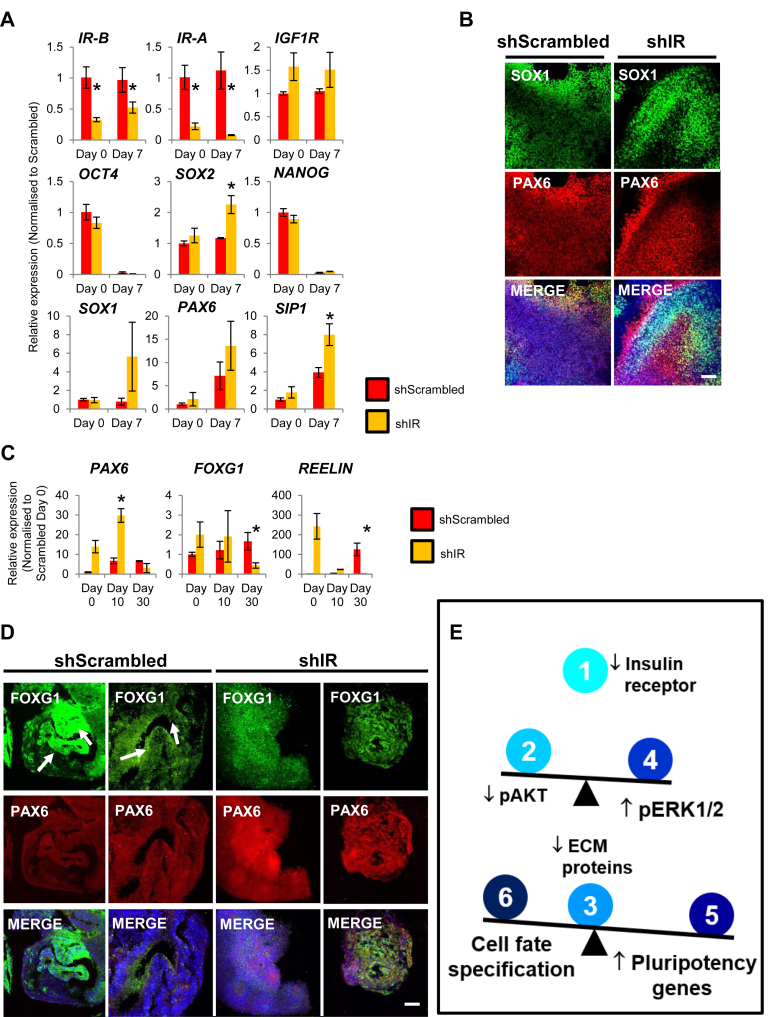
**Knockdown of IR in hPSCs perturbs neuroectoderm differentiation and subsequent formation of cerebral organoids.***A*, expression of *IR-A*, *IR-B*, *IGF1R*, *OCT4*, *SOX2*, *NANOG*, *SOX1*, *PAX6*, and *SIP1* transcripts in shScr- and shIR-hESC–derived neuroectoderm. *B*, immunostaining for SOX1 and PAX6 neuroectoderm markers in shScr- and shIR-hESCs. Scale bar: 200 μm. *C*, expression of *PAX6*, *FOXG1*, and *REELIN* transcripts in shScr- and shIR-hESC–derived cerebral organoids. All error bars indicate standard deviation of three biological replicates. *Asterisk* (∗) indicates *p* < 0.05 compared with shScr-hPSCs (Student’s *t* test). *D*, immunostaining for PAX6 and FOXG1 in shScr- and shIR-hESC–derived cerebral organoids. Cortical membrane-like structures are shown with *arrows*. Scale bar: 200 μm. *E*, summary model depicting (1) shIR-hPSCs with decreased (2) pAKT and (3) ECM protein expression, (4) elevated pERK1/2, and (5) elevated pluripotency gene expression, resulting in (6) perturbations in cell fate commitment gene expression, including that of the neural lineage. hESC, human embryonic stem cell; hPSC, human pluripotent stem cell; IR, insulin receptor.

Considering the RNA-Seq data pointed to perturbations in genes involved in nervous system development in undifferentiated hPSCs (Fig. 3, *A*–*C*), we then differentiated both shScr- and shIR-hPSCs into neuroectoderm and cerebral organoids to evaluate the effects of IR knockdown on neural lineage differentiation. QPCR analyses revealed an upregulation of neuroectodermal markers *SOX2*, *SOX1*, *PAX6*, and *SIP1* in shIR-hPSCs differentiated into day 7 neuroectoderm (Fig. 4*A*). Immunostaining then revealed that SOX1 and PAX6 proteins colocalized in shScr-hPSC–derived neuroectoderm but not in shIR-hPSC–derived neuroectoderm (Fig. 4*B* and Fig. S4).

Moving forward, we differentiated both shScr- and shIR-hPSCs into cerebral organoids using the protocol published by Lancaster *et al.* (16). We continued to observe an upregulation of *PAX6* gene expression in this advanced neural differentiation protocol (Fig. 4*C*). However, we consistently found the forebrain marker *FOXG1* and Cajal–Retzius marker *REELIN* to be significantly downregulated at the end of the cerebral differentiation (Fig. 4*C*). Immunostaining for FOXG1 in these cerebral organoids then showed that shScr-hPSC–derived cerebral organoids formed FOXG1^+^ cortical membrane-like structures (see white arrows) but shIR-hPSC–derived cerebral organoids did not (Fig. 4*D*). This suggests that the knockdown of IR in hPSCs led to aberrant neuroectoderm development, giving rise to disrupted neural gene expression and the proper formation of mature cortical structures in this cerebral organoid model.

Collectively, our data demonstrate that the knock down of IR in hPSCs (1; Fig. 4*E*) decreased pAKT (2; Fig. 4*E*) and resulted in a global decrease in ECM gene expression necessary for the hPSC niche (3; Fig. 4*E*). The knockdown also resulted in a compensatory increase in pERK1/2 (4; Fig. 4*E*), an inherent compensatory mechanism, leading to increased pluripotency marker gene expression (5; Fig. 4*E*), and perturbations in cell fate specification such as that of neural development (6; Fig. 4*E*).

## Discussion

Insulin/IGF-I signaling pathways regulate the growth and specialized functions of most mammalian tissues including pluripotent stem cells. Although a role for IGF1R has been reported (6, 7), virtually nothing is known about the significance of IR in hPSCs. Using genome-wide RNA-Seq, proteomics, and phosphoproteomics analyses of two different clones in two different hESC lines (CHB8 and H9), we demonstrated that the IR is indeed important for hPSC viability and function. Knockdown of IR marginally perturbed adaptor proteins while strongly decreasing pAKT and evoking a compensatory increase in pERK1/2 signaling. Insulin stimulation of shScr-hPSCs demonstrates that pAKT signaling is the predominant pathway activated *via* ligand binding to IR/IGF1R.

Among the nodes downstream of insulin/IGF-I signaling, both the AKT and ERK1/2 pathways (9) are considered important for pluripotency and viability of hESCs (17). In contrast to human PSCs, IR-mediated signaling showed a decrease in Erk/mTor pathways along with a decrease in Akt pathway in mouse PSCs (18), suggesting some species differences. In our studies, the severe reduction in pAKT signaling in shIR-hPSCs and a strong concomitant increase in pERK1/2 signaling is consistent with a recent report that PI3K/AKT inhibition induces pERK1/2 expression to modulate self-renewal of hPSCs (15). The decrease in pAKT signaling in our shIR-hPSCs should not be confused with the inhibition of PI3K/AKT signaling that induces endoderm differentiation because the latter takes place in the presence of a high dose of Activin A (19). Since the insulin/IGF-I signaling pathway is known to be actively involved in the maintenance of self-renewal and pluripotency of hPSCs (20) and considering the high levels of recombinant insulin/IGFs in hPSC media, one can argue that insulin/IGF-I is highly implicated in mediating the increase in pERK1/2 signaling.

Insulin/IGF-I signaling activates the MAPK pathway, and a high basal level of MAPK signaling has been reported to maintain the expression of pluripotency genes in hPSCs (21, 22). MAPK signaling is essential for the maintenance of pluripotency in both mouse and human PSCs, but, in contrast, high levels of MAPK signaling is reported to involve differentiation in mouse PSCs. The MEK/ERK signaling pathway has diverse functions with some reports indicating that it maintains pluripotency and self-renewal (17, 22) and others claiming it promotes differentiation or counters BMP-induced differentiation (23, 24). Since KOSR contains BMP-like activities, the increased pERK1/2 in shIR-hPSCs could be playing a role in inhibiting BMP signaling-induced differentiation. Increased pERK1/2 has been suggested to lead to increased differentiation (15). However, in our shIR-hPSCs, the increased pERK1/2 is tightly correlated with increased pluripotency gene expression, phosphorylation, and widespread downregulation of mesodermal and endodermal gene expression. In fact, investigation of ERK signaling for the self-renewal of hESCs reveals that ERK2 binds near *OCT4*, *SOX2*, *DPPA4*, *LIN28A*, *SALL4*, and *DNMT3B* and is necessary for the maintenance of pluripotency (10). In addition, OCT4, SOX2, and SALL4 contain putative ERK phosphorylation sites of which ERK2 was confirmed to phosphorylate OCT4 (14). The binding by ERK2 onto its target genes is reported to be phosphorylation dependent (10). Our data definitively establishes direct causality between ERK1/2 and the master pluripotency regulator OCT4 (25). These findings are corroborated by the report on the importance of ERK signaling in the maintenance of self-renewal, by phosphorylating pluripotency genes, leading to the recruitment of complexes involved in protein degradation, reduced transcriptional activity, or protein stability (26, 27). The increase in *OCT4* gene expression could in turn upregulate the expression of other downstream pluripotency genes to curb undesired differentiation and to maintain the hPSC state.

OCT4 and SOX2 are at the core of the self-renewal and pluripotency network of hPSCs (2). UTF1, a target gene of OCT4 and SOX2, functions to regulate differentiation *via* the tight control of bivalent genes. Other pluripotency factors such as DPPA4 inhibit ESC differentiation; DNMT3B is a DNA methyltransferase that regulates developmental potential of PSCs, whereas LIN28A potentiates insulin/PI3K signaling *via* the repression of let-7. Therefore, the increased expression of these pluripotency genes appears to counter the effects of IR knockdown *via* diverse mechanisms that serve to maintain the self-renewal and pluripotent state of hPSCs.

Among the pluripotency genes, OCT4, SOX2, and NANOG are placed at the top of the hierarchy. Increased levels of OCT4 and SOX2 are known to suppress mesodermal and endodermal genes (28). In agreement with this, many germ layer specification genes, primarily endodermal and mesodermal, were strikingly downregulated in hPSCs with reduced insulin receptors. In these shIR-hPSCs, which exhibit perturbed pluripotency *versus* cell fate commitment balance, there is an initial increase in the expression of *PAX2*, *GBX2*, *CHRD*, *NES*, and numerous *HOX* genes. The increased expression of SOX2 that can initiate the neural cell fate (29) likely explains this phenomenon. Overall, the cause for imbalance in germ layer specification genes is likely due to an increase in phosphorylation coupled with total protein expression of numerous pluripotency genes.

The ECM is necessary for survival and proliferation of epithelial cells such as hPSCs. Knockdown of IR in hPSCs resulted in a global downregulation of ECM proteins. To our knowledge, there is limited evidence to date linking the role of IR signaling to ECM formation (30). ERK1/2 signaling is apparently required for cell adhesion to facilitate hPSC clonogenicity (23). In addition, increased pERK1/2 signaling reduces stem cell differentiation (31). Collectively, this suggests that the increased pERK1/2 in shIR-hPSCs is a feedback response to counter shIR-mediated loss of ECM proteins, to improve cellular adhesion, hPSC self-renewal, and survival and to decrease differentiation.

Our RNA-Seq data on shIR-hPSCs first revealed distinct effects on nervous system development. Further differentiation of these shIR-hPSCs into neuroectoderm and cerebral organoids then demonstrated various aberrant signatures, consistent with the importance of insulin signaling during brain development (32, 33). Since the Sox1 to Pax6 switch is involved in radial glia progression (34), the dysregulation of *SOX1* and *PAX6* gene expression and protein localization could affect the specialization of neural cells. *FOXG1* is a critical transcription factor playing pleiotropic functions during brain development (35). It suppresses premature cortical cell fate (36) and maintains the proliferative state of neurons (37). The gene dosage of *FOXG1* can account for the various types of FOXG1 syndrome (38). Given that *FOXG1* is known to be directly regulated by AKT signaling (37), the loss of IR can partly contribute to its reduced expression, thereby leading to the abnormal structures in the cerebral organoids.

In summary, our follow-up study (18) using genome-wide RNA-Seq, proteomics and phosphoproteomics analyses, and its validation in various clones of two different hESC lines (CHB8 and H9) demonstrates the importance of IR-mediated pAKT signaling in hPSCs, without which ECM formation, which is critical for cellular attachment, is severely attenuated. An inherent compensatory mechanism in the form of upregulated pERK1/2 signaling leads to increased pluripotency gene expression and a perturbation of the balance between pluripotency *versus* cell fate commitment (Fig. 4*E*). Overall, this study links IR-mediated signaling to the pluripotent function of hPSCs and the proper regulation of cell fate specification, including that of the neural lineage. Future experiments are warranted to elucidate ERK1/2-bound targets in shIR-hPSCs to potentially reveal direct regulatory relationships with germ layer–specific genes.

## Experimental procedures

### Cell culture

CHB8 (Daley lab) and H9 (WiCell) hESCs stably transfected with shIR plasmids were cultured in mTeSR1 media supplemented with 1 μg/ml puromycin (28). Two different shIR plasmids were used to generate independent IR knocked down clones in CHB8 and H9 hESCs. For stimulation assays, hPSCs were grown in DMEM/F-12 + 0.5% BSA + 10 ng/ml Activin + 12 ng/ml FGF2 (3) for 24 h before being stimulated with 0, 1, 10, or 100 nM human insulin for 5 min. TCS ERK 11e (Tocris Bioscience) was used to inhibit ERK2, whereas SCH772984 (Selleck Chemicals) was used to inhibit ERK1/2. hPSCs were treated with the ERK1/2 inhibitors for 72 h. hESCs used were tested mycoplasma negative.

For neuroectoderm differentiation, hESCs were cultured following the protocol established in a previous study by Chng *et al.* (29). For cerebral organoid differentiation, hESCs were grown to 90% confluency, dissociated into single cells using TrypLE Express Enzyme (Thermo Fisher), replated in TeSR-E8 media with 50 μM Y-27632, and seeded into ultralow-attachment 96-well plates (12,000 cells per well) (Corning Costar). After 24 h (designated as D0), the embryoid bodies were differentiated into neuroectoderm, neural progenitor, and cerebral organoids using a previously established protocol (16).

### Teratoma and *in vitro* differentiation assays

The teratoma assay has been described previously (39). The embryoid body formation and *in vitro* differentiation assay to ascertain differentiation potential has been reported previously (40). Antibodies used are provided in Table S6.

### qRT-PCR, Western blot, immunostaining, and chromatin immunoprecipitation analyses

Methods for qRT-PCR, Western blot, and immunostaining analyses have been described previously (41). The method for ChIP has been described previously (10, 28, 42). Western blot bands were quantitated using TotalLab Quant or LI-COR Image Studio Lite. All error bars represent standard deviation of three biological replicates. A *p* value <0.05 indicates statistical significance by two-sided Student’s *t* test. Primers and antibodies used are provided in Table S6.

### Luciferase assay

*OCT4*, *SOX2*, and *DPPA4* genomic regions bound by ERK2 (10) were cloned into pGL4.23 or pGL4.10 luciferase vectors using NheI and HindIII restriction enzyme sites. Primers used for cloning are provided in Table S6.

### ELISA assay

Human insulin ELISA assay (Mercodia) was performed by the Joslin Specialized Assay Core.

## RNA-Seq and MS data availability

RNA-Seq was performed at the Broad Institute. Raw data have been uploaded to GEO with accession number GSE60328. MS was performed in the Environmental Molecular Science Laboratory at Pacific Northwest National Laboratory. Raw data have been deposited in MassIVE (https://massive.ucsd.edu/) with accession number MSV000085298. See Supplementary Information for details.

## Supporting information

This article contains supporting information.

## Conflict of interest

The authors declare that they have no conflicts of interest with the contents of this article.
