# Supplementary material for: Defective insulin receptor signaling in hPSCs skews pluripotency and negatively perturbs neural differentiation
Source: J Biol Chem. 2021 Mar 3;296:100495. doi: 10.1016/j.jbc.2021.100495 (PMC8050001; doi:10.1016/j.jbc.2021.100495)

SUPPLEMENTARY INFORMATION

SUPPLEMENTARY MATERIALS AND METHODS

RNA-Seq

cDNA Library Construction

Total RNA was extracted using RNeasy Mini Kit according to the manufacturer's protocol. Total RNA was quantified using theQuant-iT™ RiboGreen® RNA Assay Kit and normalized. Libraries were prepared with an automated variant of the Illumina TruSeq™ mRNA Sample Preparation Kit that preserves strand orientation of the RNA transcript. This method uses oligo-dT beads to select mRNA from the total RNA sample, followed by heat fragmentation and cDNA synthesis from the RNA template. The resulting cDNA then goes through library preparation (end repair, base 'A' addition, adapter ligation, and enrichment) using indexed adapters for multiplexing. After enrichment, the libraries were quantified by qPCR using the KAPA Library Quantification Kit for Illumina Sequencing Platforms and then equimolar pooled.

Illumina Sequencing

Pooled libraries were normalized and denatured using 0.2N NaOH prior to sequencing. Flowcell cluster amplification and sequencing were performed according to the manufacturer's protocols on an Illumina HiSeq 2500 at the Broad Institute Specialized Service Facility. Each run generated 76bp paired-end reads with 8bp barcodes. Two

biological replicates were sequenced for shScr- and shIR-CHB8 hESCs. Base calling, de-multiplexing and data aggregation were performed with Picard Tools v1.74.

Differential Expression Analysis of RNA-Seq

Reads were mapped to the human reference genome (build 19) using TopHat v2.0.10 [1] with the following options: --no-coverage-search; --num-threads 8; ---transcriptome-index with a TopHat transcriptome index built from GENCODE v19. Gene expression levels were estimated with Cufflinks v2.2.0 [2]. Cuffdiff was used to compute the log2 fold change between conditions and assign p values based on simulated draws from a beta negative binomial model. Expression differences were deemed significant if Cuffdiff gave a p value below 0.05.

Mass spectrometry (MS)-based quantitative global proteomics and phosphoproteomics

Protein digestion and TMT labeling of peptides

Cell pellets were lysed in 100μL lysis buffer containing 8M urea, 75mM NaCl in 100mM NH_4HCO_3 (pH 7.8), 10mM NaF, phosphatase inhibitor cocktail 2 (Sigma, P 5726) and cocktail 3 (Sigma, P0044), and the cOmplete Ultra protease inhibitor (Roche, 05 892 791 001). Protein concentrations were determined by BCA assay (Pierce). Proteins were reduced with 5mM DTT (dithiothreitol) for 1h at 37°C, and were subsequently alkylated

with 10mM iodoacetamide. Samples were diluted 2-fold with 50mM NH4HCO3 and digested with trypsin (Promega, V5113) at a ratio of 1:50 trypsin:protein (w/w) for 4h at 37°C, diluted by 4-fold, and subjected to a second treatment with trypsin and incubation at room temperature overnight (~16h). The digest was acidified with trifluoroacetic acid (TFA) to pH ~2.5. Tryptic peptides were desalted on reversed phase C18 SPE columns (SUPELCO Discovery, 50mg, 52601-U) and dried using SpeedVac.

Desalted peptides were labeled with 6-plex tandem mass tags (TMT) reagents according to the manufacturer's instructions (Pierce, Rockford, IL). 100μg peptides from each sample were dissolved in 30μL of 0.5M triethylammonium bicarbonate (TEAB) (pH 8.5) solution and labeling reagent in 70μL of ethanol was added for each channel. After 1h incubation, 8μL of 5% hydroxylamine was added to hydrolyze excess reagents by additional 15min incubation. Differential labeled peptides from all six channels (126, 127, 128, 129, 130, 131) were pooled, acidified by 10% TFA, and desalted again using C18 SPE columns.

Offline fractionation of peptides and enrichment of phosphopeptides

TMT-labeled peptides were fractionated using a RP XBridge C18 column from Waters (250mm × 4.6mm column containing 5μM particles) using Agilent 1200 HPLC System. The sample loaded onto the C18 column was washed for 9min with 5% of solvent B (10mM TEAB, pH 7.5, 90% ACN), followed by 10min solvent A (10mM TEAB, pH 7.5)

equilibration before an 109min LC gradient. The LC gradient started with a linear increase of solvent A to 10% B in 3min, then linearly increased to 30% B in 86min, 10min to 42.5% B, 5min to 55% B and another 5min to 100% solvent B. The flow rate was 0.5mL/min. A total of 96 fractions were collected into a 96 well plate through the LC gradient. The high pH RP fractions were combined into 24 fractions using the concatenation strategy reported in our previous study [3]. For global proteome analysis, 20% of each concatenated fraction was dried down and re-suspended in 0.1% TFA to a peptide concentration of 0.15μg/μL. The rest of the concatenated fractions (80%) were further concatenated into 4 fractions and subjected to immobilized metal affinity chromatography (IMAC) for phosphopeptide enrichment. Magnetic $Fe3+$-NTA-agarose beads were prepared using the Ni-NTA-agarose beads (Qiagen, Valencia, CA,) following the protocols reported [4].

Briefly, peptides (~100μg) were reconstituted in 300μL IMAC binding/wash buffer (80% MeCN, 0.1% TFA) and incubated for 30min with 75μL of the 5% bead suspension. After incubation, the beads were washed 4 times each with 300μL of wash buffer. Phosphorylated peptides were eluted from the beads using 50-75μL of 1:1 ratio of acetonitrile to 2.5 % ammonia in 2mM (pH 8) phosphate buffer (v/v) after incubating for 1.5min. Samples were acidified and concentrated, then were reconstituted to 30μL with 0.1% TFA for LC-MS/MS analysis.

LC-MS/MS analysis

All peptide samples were analyzed using an automated home-built constant flow nanoLC system (Agilent) coupled to an LTQ-Orbitrap-Velos mass spectrometer (Thermo Scientific). The nano LC system for phosphoproteomics analysis has an online 4-cm x 360µm o.d. x 150µm i.d. C18 SPE column (5-µm Jupiter C18, Phenomenex, Torrence, CA) to desalt each phosphopeptide sample (20µL), which is connected to a home-made 60-cm x 360µm o.d. x 50µm i.d. capillary column (3-µm Jupiter C18, Phenomenex, Torrence, CA). Mobile phase flow rate was 100nL/min and consisted of 0.1M acetic acid in water (A) and 0.1M acetic acid in 70:30 (v/v) acetonitrile:water (B) with a gradient profile as follows (min:%B); 0:0, 5:10, 140:35, 160:60, 165:90, 170:90. For global proteome analysis, an on-line 4-cm x 360µm o.d. x 150µm i.d. SPE column packed with 3.6-µm Aeris Widepore XB-C18 and a 35-cm x 360µm o.d. x 75µm i.d. fused-silica capillary analytical column (3µm Jupiter C18) were used. Mobile phases consisted of 0.1% formic acid in water (A) and 0.1% formic acid acetonitrile (B) operated at 300nL/min with a gradient profile as follows (min:%B); 0:5, 2:8, 20:12, 75:35, 97:60, 100:85.

The LTQ Orbitrap Velos mass spectrometer was operated in the data-dependent mode acquiring higher-energy collisional dissociation (HCD) scans (R=7,500, 5 × 104 target ions) after each full MS scan (R=30,000, 3 × 106 target ions) for the top ten most abundant ions within the mass range of 300 to 1800 m/z. An isolation window of 2.5 Th was used to isolate ions prior to HCD. All HCD scans used a normalized collision energy of 45 and

a maximum inject time of 1000ms. The dynamic exclusion time was set to 60s and charge state screening was enabled to reject unassigned and singly charged ions.

Data analysis

MS/MS raw data were converted to .dta files using an in-house software DeconMSn. Peptides were identified from MS/MS spectra using database searching algorithm MS-GF+ v9949 [5] against the human protein database (20249 entries) downloaded from Uniprot (downloaded on April 2012). Static modification of 6-plex TMT (229.1629 Da) on lysine residue and N-termini of peptides, carbamidomethylation of cysteine residues (57.0215 Da) and dynamic oxidation of (15.9949 Da) methionine were applied during the database search. Both fully and partially tryptic peptides were considered with 2 missed cleavages allowed. The mass tolerance for precursor ions was 50 ppm and fragmentation tolerance for HCD were 0.05 Da. All peptides were identified with <0.1% False Discovery Rate by using a MS-Generating Function Score (MS-GF) <1E-10 and a decoy database searching strategy [6]. The reporter ion intensities for each channel in each biological condition were extracted with an in-house tool MASIC for quantification. For a given protein, the reporter ion intensities in each channel were summed for all identified spectra. Three biological replicates for shScr- and shIR-hPSCs (CHB8 and H9) were analysed. Statistical analysis of significance was performed using the software tool DAnTE [7] to identify proteins with significant abundance changes. p value is calculated based on the ANOVA (analysis of variance) test and the corresponding q value denotes the false discovery rate [8].

Gene ontology (GO) overrepresentation analysis

The overrepresentation of GO biological process (BP) categories was assessed using DAVID [9].

Data visualization

Heat maps were generated in R with the ggplot2 library [10]. Rows were ordered by fold change (taken as the ratio of the average knockdown level to the average control level) and then converted to per-row z-scores for color mapping.

SUPPLEMENTARY REFERENCES

1. Trapnell C, Pachter L, Salzberg SL. TopHat: discovering splice junctions with RNA-Seq. Bioinformatics. 2009;25(9):1105-11. Epub 2009/03/18. doi: 10.1093/bioinformatics/btp120. PubMed PMID: 19289445; PubMed Central PMCID: PMC2672628.

2. Trapnell C, Williams BA, Pertea G, Mortazavi A, Kwan G, van Baren MJ, et al. Transcript assembly and quantification by RNA-Seq reveals unannotated transcripts and isoform switching during cell differentiation. Nat Biotechnol. 2010;28(5):511-5. Epub 2010/05/04. doi: 10.1038/nbt.1621. PubMed PMID: 20436464; PubMed Central PMCID: PMC3146043.

3. Wang Y, Yang F, Gritsenko MA, Clauss T, Liu T, Shen Y, et al. Reversed-phase chromatography with multiple fraction concatenation strategy for proteome profiling of human MCF10A cells. Proteomics. 2011;11(10):2019-26. Epub 2011/04/19. doi: 10.1002/pmic.201000722. PubMed PMID: 21500348; PubMed Central PMCID: PMC3120047.

4. Ficarro SB, Adelmant G, Tomar MN, Zhang Y, Cheng VJ, Marto JA. Magnetic bead processor for rapid evaluation and optimization of parameters for phosphopeptide enrichment. Anal Chem. 2009;81(11):4566-75. doi: 10.1021/ac9004452. PubMed PMID: 19408940; PubMed Central PMCID: PMC2692042.

5. Kim S, Mischerikow N, Bandeira N, Navarro JD, Wich L, Mohammed S, et al. The Generating Function of CID, ETD, and CID/ETD Pairs of Tandem Mass Spectra: Applications to Database Search. Mol Cell Proteomics. 2010;9(12):2840-52. doi: DOI 10.1074/mcp.M110.003731. PubMed PMID: ISI:000284882100020.

6. Qian WJ, Liu T, Monroe ME, Strittmatter EF, Jacobs JM, Kangas LJ, et al. Probability-based evaluation of peptide and protein identifications from tandem mass spectrometry and SEQUEST analysis: the human proteome. J Proteome Res. 2005;4(1):53-62. PubMed PMID: 15707357.

7. Polpitiya AD, Qian WJ, Jaitly N, Petyuk VA, Adkins JN, Camp DG, 2nd, et al. DAnTE: a statistical tool for quantitative analysis of -omics data. Bioinformatics. 2008;24(13):1556-8. Epub 2008/05/06. doi: 10.1093/bioinformatics/btn217. PubMed PMID: 18453552; PubMed Central PMCID: PMC2692489.

8. Storey JD. The positive false discovery rate: a Bayesian interpretation and the *q*-value. Ann Statist. 2003;31(6):2013-35.

9. Huang da W, Sherman BT, Lempicki RA. Systematic and integrative analysis of large gene lists using DAVID bioinformatics resources. Nat Protoc. 2009;4(1):44-57. Epub 2009/01/10. doi: 10.1038/nprot.2008.211. PubMed PMID: 19131956.

10. Wickham H. ggplot2: elegant graphics for data analysis. Springer New York. 2009.

SUPPLEMENTARY FIGURE LEGENDS

Fig S1 (related to Fig 1). Knock down of IR in CHB8 and H9 hESCs perturbs insulin signaling pathway. Expression of *IR-B*, *IR-A* and *IGF1R* transcripts in shScr- and shIR- (A) CHB8 and (B) H9 hESCs. At least two independent experiments have been performed. All error bars indicate standard deviation of three biological replicates. Asterisk (*) indicates $p<0.05$ compared to shScr-hPSCs (Student's t test). Western blot analyses for IRS-1, IRS-2, p85α and ACTIN protein levels in shScr- and shIR- (C) CHB8 and (D) H9 hESCs. (E) Western blot analyses for IR, IGF1R, IRS-1, IRS-2, pAKT, tAKT, pERK1/2, tERK1/2 and ACTIN protein levels in additional independent IR knocked down clones in H9 hESCs generated using different shRNA constructs.

Fig S2 (related to Fig 2). shIR-hPSCs remain pluripotent and exhibit increased expression of pluripotency genes. (A) Teratoma assay demonstrates that shScr- and shIR-hPSCs can differentiate into the three germ layers *in vivo*. Scale bar: 100μm. (B) Immunostaining for ectoderm markers SOX1 and PAX6, extraembryonic marker SOX7, and endoderm markers SOX17 and FOXA2 in differentiated shScr- and shIR-H9 hESCs. Scale bar: 50μm. (C) Table depicting statistically significant up-regulated pluripotency genes (RNA-Seq data) in shIR-CHB8 hESCs. (D) Table depicting statistically significant up-regulated pluripotency proteins (MS data) in shIR-CHB8 hESCs. (E) Heat map of MS analyses showing expression of proteins involved in pluripotency from shScr- and shIR-H9 hESCs (up-regulation in red, down-regulation in blue). (F) Table depicting statistically significant up-regulated pluripotency proteins (MS

data) in shIR-H9 hESCs. (G) Western blot analyses for OCT4, SOX2, DPPA4, LIN28 and ACTIN protein levels in shScr- and shIR-H9 hESCs, and (H) Western blot analyses of proteins in additional independent IR knocked down clones in H9 hESCs generated using different shRNA constructs.

Fig S3 (related to Fig 3). Knock down of insulin receptor in hPSCs causes global down-regulation of ECM proteins. (A) Heat map of RNA-Seq analyses of expression of ECM genes from shScr- and shIR-CHB8 hESCs (up-regulation in red, down-regulation in blue). Heat map of MS analyses showing expression of ECM proteins from shScr- and shIR- (B) CHB8 and (C) H9 hESCs (up-regulation in red, down-regulation in blue). (D) Expression of *FN1*, *COL1A1* and *KRT19* transcripts in shScr- and shIR-hESCs. All error bars indicate standard deviation of three biological replicates. Asterisk (*) indicates $p<0.05$ compared to shScr-hPSCs (Student's t test). (E) Western blot analyses for FN1, COL1, P4HB and ACTIN protein levels in shScr- and shIR-hESCs.

Fig S4 (related to Fig 4). Knock down of IR in hPSCs perturbs neuroectoderm differentiation. Immunostaining for SOX1 and PAX6 neuroectoderm markers in additional independent shScr- and shIR-hESCs. Scale bar: 200μm.

SUPPLEMENTARY TABLE LEGENDS

Table S1. RNA-Seq performed on shIR-CHB8 hESCs.

Table S2. Proteomics performed on shIR-CHB8 hESCs.

Table S3. Proteomics performed on shIR-H9 hESCs.

Table S4. Phosphoproteomics performed on shIR-H9 hESCs.

Table S5. Phosphoproteomics performed on shIR-CHB8 hESCs.

Table S6. Primers and antibodies used.

Figure S1: Teo et al.,

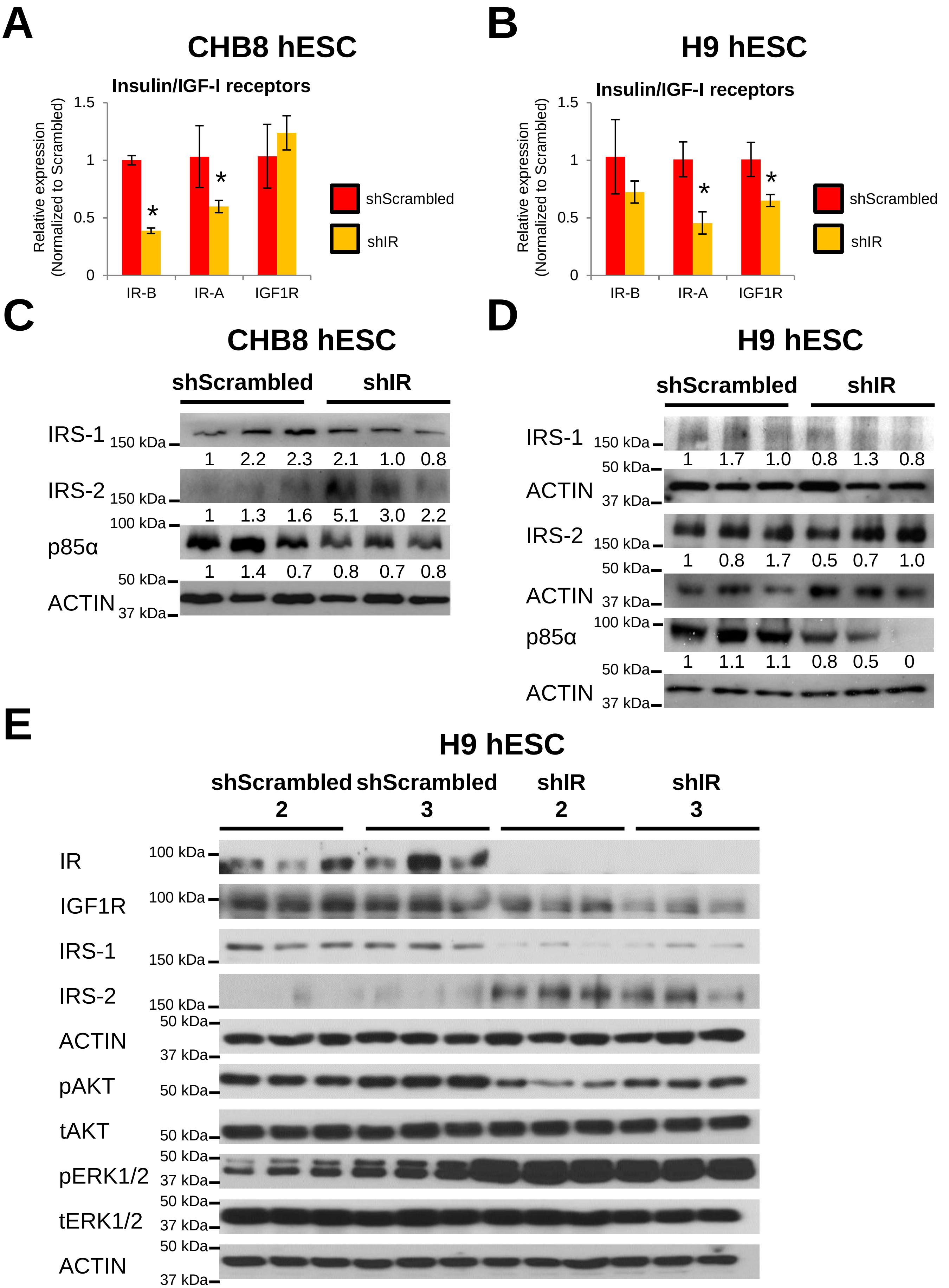

Figure S2: Teo et al.,

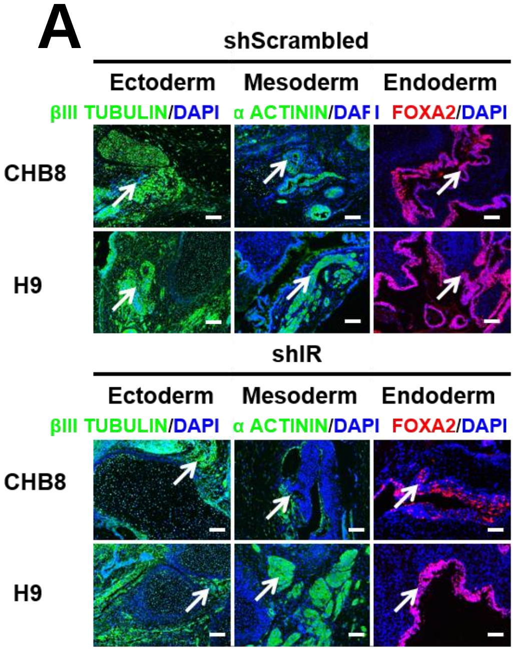

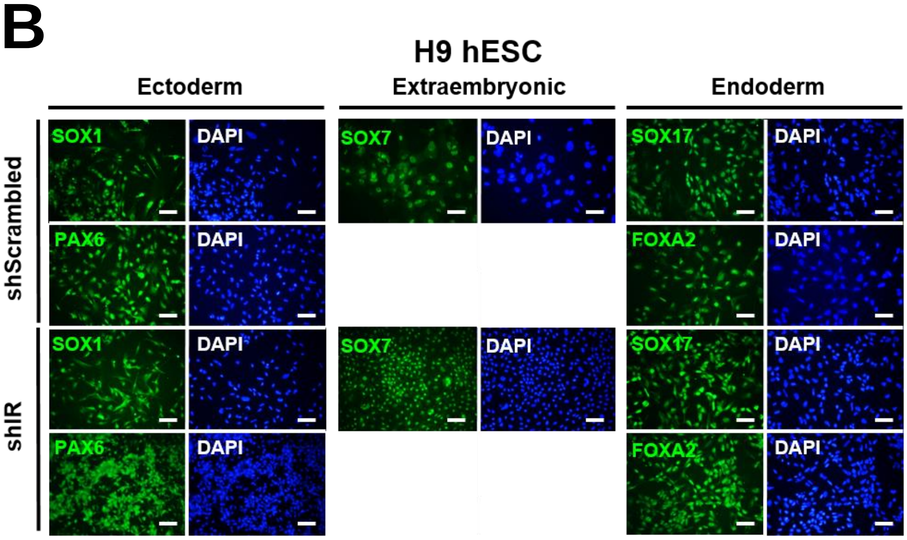

C

CHB8
RNA-Seq ($p < 0.05$)

Gene	Fold change
NR6A1	3.32
NR5A2	2.47
SFRP2	2.01
LIN28A	1.69
SOX2	1.60
MYCL	1.60
MYCN	1.52
PODXL	1.52
CRABP2	1.48
SALL4	1.47
GABRB3	1.41
DNMT3B	1.32
TEAD2	1.31

D

CHB8 MS ($p < 0.05$)

Protein	Ratio (shIR/shScr)
NR6A1	1.71
DPPA4	1.41
TEAD2	1.40
PODXL	1.39
SALL4	1.27
LN28A	1.25
SOX2	1.21
TF2L1	1.20

E

H9 MS

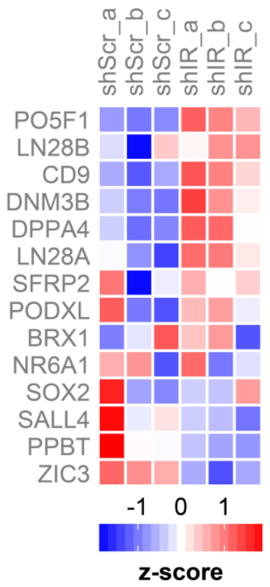

F

H9 MS ($p < 0.05$)

Protein	Ratio (shIR//shScr)
PO5F1	2.11
CD9	1.46
DNM3B	1.36
DPPA4	1.33

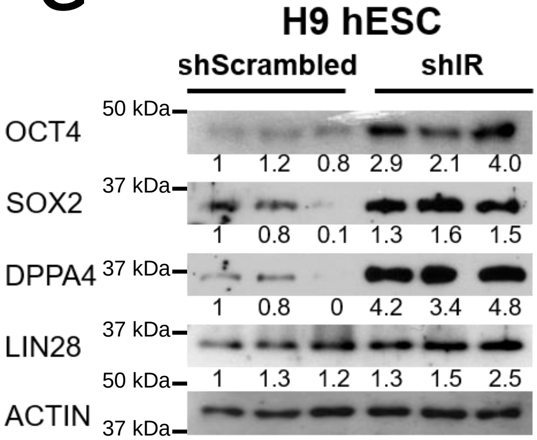

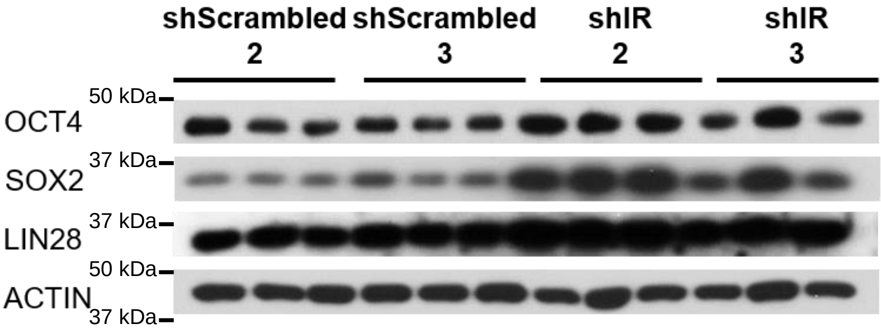

Figure S3: Teo et al.,

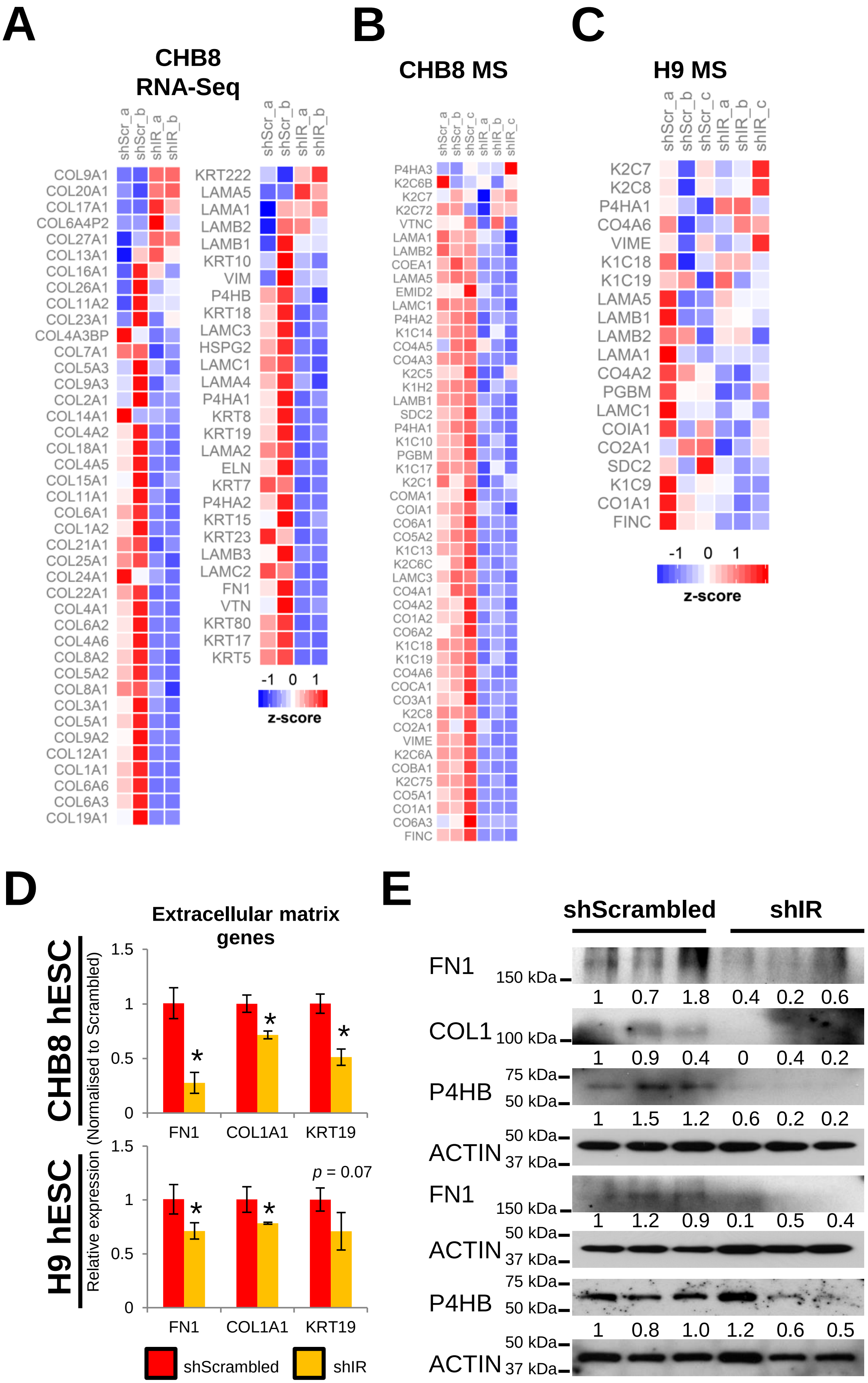

Figure S4: Teo et al.,

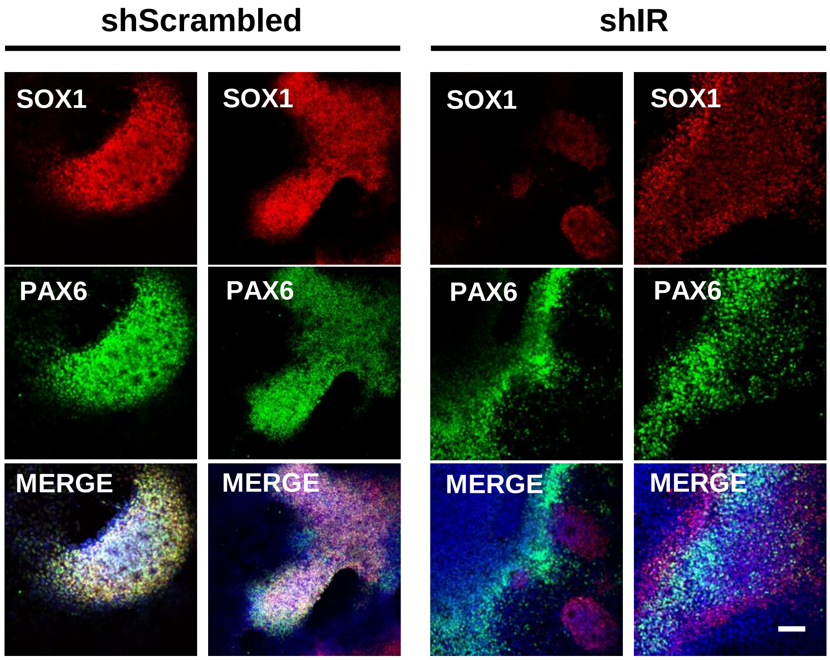

Supplement: Supplemental Figures S1–S4 [file mmc7.pdf]
